# Exploring Bikeability in a Suburban Metropolitan Area Using the Active Commuting Route Environment Scale (ACRES)

**DOI:** 10.3390/ijerph110808276

**Published:** 2014-08-15

**Authors:** Lina Wahlgren, Peter Schantz

**Affiliations:** 1 The Research Unit for Movement, Health and Environment, The Swedish School of Sport and Health Sciences, GIH, SE-114 86 Stockholm, Sweden; E-Mail: peter.schantz@gih.se; 2 Department of Health Sciences, Mid Sweden University, SE-831 25 Östersund, Sweden

**Keywords:** active transport, bicycle commuting, bikeability, perception, route environment, suburban area

## Abstract

*Background and Aim:* Commuting by bicycle could contribute to public health, and route environments may influence this behaviour. Therefore, the aim of this study is to assess the potential associations between appraisals of the overall route environment as hindering or stimulating for bicycle commuting, with both perceptions of commuting route environmental factors in a suburban area and background factors. *Methods:* The Active Commuting Route Environment Scale (ACRES) was used for the assessment of bicycle commuters’ perceptions and appraisals of their route environments in the suburban parts of Greater Stockholm, Sweden. A simultaneous multiple regression analysis was used to assess the relationship between the outcome variable whether the overall route environment *hinders or stimulates* bicycle commuting and environmental factors (e.g., *exhaust fumes*, *speeds of motor vehicles*, *greenery*), as well as background factors (*sex*, *age*, *education*, *income*) as predictor variables. *Results and Conclusions:* The results indicate that in suburban areas, the factors aesthetics, greenery and bicycle paths seem to be, independently of each other, stimulating factors for bicycle commuting. On the other hand, flows of motor vehicles, noise, and low “directness” of the route seem to be hindering factors. A comparison of these results with those obtained from an inner urban area points to the importance of studying different types of built-up areas separately.

## 1. Introduction

Physical activity has several considerable health benefits [1]. However, in the industrialized world, the previous century’s developments have resulted in a general lifestyle of physical inactivity. Therefore, increasing the level of physical activity is a public health concern [2]. Active commuting—walking and cycling to get to and from work—could constitute a way for people to be physically active (for reviews, see [3,4]). An additional benefit of such a behaviour is a better local traffic environment with potentially fewer traffic emissions and less noise, and thereby positive generalized health effects in the population.

It is not easy to acquire knowledge concerning factors of importance for a behaviour that can be dependent on so many aspects. For example, one may cycle or not owing to reasons of economy, public transport availability, car and cycle parking conditions, distances to destinations, route environments, motivation for physical exercise, physical capacity, self-efficacy, and so forth. Owing to the multiplicity of possible causative ingredients, it is, in our perception, necessary to try to gain knowledge about each one of them *per se*, with the aim of integrating them in future systemically oriented analyses of predictors of cycling behaviour.

Our focus here is on gaining knowledge about how different specific environmental variables in the route environments can affect overall appraisals of whether they inhibit or stimulate cycling. This is due to that route environments can be crucial for the decision to cycle or not to cycle. Given that a cycling behaviour exists, route environments may also affect the number of trips, their duration and distances, as well as the persistence of a particular cycling behaviour with the passage of time. We have termed these types of effects as the *bikeability* of route environments [5].

Another reason for this focus is well-being. It is part of what constitutes health [6]. It is therefore of interest to acquire knowledge about how route environments affect environmental well-being when cycling [7]. It has been hypothesized that whether or not a route environment hinders or stimulates cycling affects it [7], and recent empirical evidence lends support to such a linkage (Schantz *et al.*, personal communication).

Thus, two different outcomes—cycling behaviour and environmental well-being when cycling—prompt studies to determine which specific variables are important for appraisals of hindering or stimulating route environments for cycling. Such knowledge is of value *per se*, as well as for, e.g., politicians, planning professionals, and advocacy groups aiming at creating better conditions for cycling.

Route environments are often complex settings, with a large number of variables involved. They are therefore difficult to study. However, with the aim of developing this field of knowledge, principally different types of important strategies, that make use of photographs or real world settings, have been developed. One of them deals with holistic ratings of photographs of route settings (e.g., [8]). Others make use of different forms of stated preferences for different ingredients in the route environments (e.g., [9]). In the first type of study, it is difficult to state and rate the importance of each of the many environmental variables involved, whereas in the other type of study, particular aspects can be evaluated, but not in relation to a wholeness of route environmental factors that may be of importance. A third type of research strategy is based on route choice analyses in real world settings (e.g., [10]). In principle, this can be a fruitful path for analyses. However, in order to pinpoint specific variables of importance, a wide variability of route settings is needed. Furthermore, the route choice must be based on knowledge, among both cyclists and researchers, concerning possible route alternatives and their relevant environmental characteristics. These conditions are very difficult to fulfill. Furthermore, if objective descriptive data coupled to routes are used, the poor agreement between objective data and perceptions adds to the difficulty of interpreting such studies [11,12,13].

The mentioned research strategies have both strengths and weaknesses. Depending on the purpose of a study, they may be very useful. However, they do not permit, or at least greatly complicate, combining experiences of the wholeness of real world route settings with the possibility of isolating the importance of single environmental variables for overall appraisals. Another principal concern refers to the nature of the outcomes often used. For example, different levels of stated preferences cannot differentiate between whether all alternatives are attractive to various degrees or some of them are neutral or repulsive (*cf.* [9]). In our opinion, such distinctions can be very important for the interpretation of route settings in relation to both cycling behaviour and environmental well-being when cycling.

Given that it is a rather new research field adds to a status of, in our mind, a fragmented, meagre and uncertain or limited knowledge base. We have therefore recognized a benefit in developing a complementary research strategy, and for that purpose the Active Commuting Route Environment Scale (ACRES) was created [14]. The ACRES assesses bicyclists’ perceptions and appraisals of their self-chosen commuting route environments based on a spatial match between the cycling behaviour and the environment in which the cycling takes place. The ACRES includes overall outcome variables (*hinders or stimulates* and *traffic: unsafe or safe*) and environmental predictor variables, such as exhaust fumes, hilliness and velocity of motor vehicles. The ACRES has 15-point response scales that allow finer distinctions, correlation assessments and multiple regression analyses.

After methodological surveys established the scale as having considerable criterion-related validity and reasonable test-retest reproducibility [5,14], we studied how bicycle commuting routes in inner urban and suburban parts, respectively, of the metropolitan area of Greater Stockholm, Sweden, were rated. Distinctly different route environmental profiles were noted for these areas. We also found that the suburban areas, compared to the inner urban areas, were rated as being more stimulating and safe to cycle in from a traffic point of view [5].

We then initiated studies with the aim of isolating the environmental predictor variables that might possibly explain differences in ratings of whether route environments are appraised as inhibiting or stimulating for commuter cycling. Our first study involved the inner urban area of Greater Stockholm. In a multiple linear regression analysis, we noted that about 40% of the variance of the outcome variable *hinders or stimulates* was explained by five environmental predictors: two with a stimulating effect, namely, *ugly or beautiful* and *greenery*, and three with an inhibiting effect, namely, *course of the route*, *exhaust fumes* and *congestion: all types of vehicles* [15].

Given the finding of distinctly different route environmental profiles of inner urban areas and suburban areas, respectively [5], and other major differences in the nature of these areas as well as population density [16], it is possible that the role of environmental variables may differ between them. Therefore, the aim of this study is to expand the analyses of the role of environmental predictor variables to the suburban metropolitan areas of Greater Stockholm.

## 2. Methods

### 2.1. Procedure and Participants

This study is part of a research project called the Physically Active Commuting in Greater Stockholm (PACS; http://www.gih.se/pacs). Active commuters; pedestrians and bicyclists, were recruited to the PACS-project, by advertising in two large morning newspapers in Stockholm (Dagens Nyheter and Svenska Dagbladet) towards the end of May and early June 2004. Inclusion criteria were: (a) being at least 20 years old; (b) living in Stockholm County, excluding the municipality of Norrtälje; and (c) walking and/or cycling the whole way to one’s place of work or study at least once a year. The place of work or study is referred to as place of work, unless stated otherwise. It was emphasized in the invitation to participate that people with short commuting distances were also welcome to participate. The purpose of including people with a less frequent active commuting behaviour and/or short route distance was to include diverse commuting behaviours.

**Figure 1:**
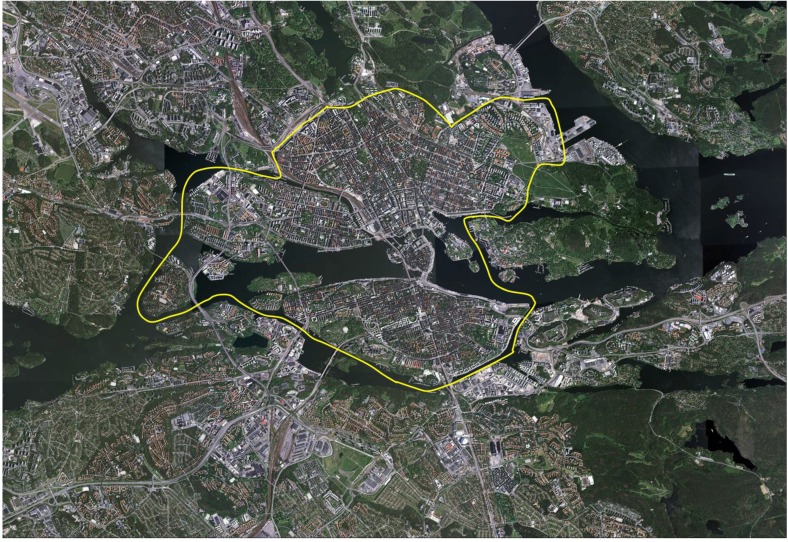
Aerial view from 2005 over the more central parts of Greater Stockholm, Sweden. The yellow line distinguishes the inner urban and the suburban, as well as rural parts, of the area. For a description of the characteristics of the suburban area, see Methods. (Copyright is granted from Lantmäteriverket, Gävle, Sweden in 2011).

The advertisement led to 2148 active commuters volunteering to participate. A first questionnaire, called the Physically Active Commuting in Greater Stockholm Questionnaire (PACS Q1), was sent to the participants in September 2004. The response frequency was 94% (n = 2010). A second questionnaire, the PACS Q2, was sent to 1978 participants in May 2005. May is the peak bicycle-commuting period of the year. The response frequency was 92% (n = 1819). The questionnaires and prepaid return envelopes were sent home to each participant by post. A maximum of three reminders were sent out. No incentives were provided for participation. Some participants were excluded in the second part of the study because they did not wish to proceed as participants. The participants were bicyclists, pedestrians or dual-mode commuters, *i.e.*, individuals who sometimes walk and sometimes cycle. They commuted in the inner urban or suburban–rural areas of Greater Stockholm, or in both of these areas (see Figure 1). The suburban–rural areas are referred to as suburban areas, unless stated otherwise. We have only used data on bicycle-commuting in the suburban areas in this study. We have previously shown that commuting in both areas does not generally affect ratings as compared to only cycling in one of these areas [5]. In this study, we have therefore combined the two groups: those who bicycle-commuted in both inner urban and suburban areas and those who bicycle-commuted in only a suburban area, and used the data from the ratings of suburban areas. Initially, 1107 participants (women, n = 701, 63%) were included in the analyses after cleansing and editing the data. For further descriptive background factors of the participants, see Table 1. The Ethics Committee of the Karolinska Institute approved the study. The participants gave their informed consent.

**Table 1:** Background factors of participants (n = 1090–1107).

Background Factor		
Females *, %		63
Age in years *, mean ± SD		48.4 ± 10.3
Weight in kg, mean ± SD		69.8 ± 11.0
Height in cm, mean ± SD		172.4 ± 8.9
Body mass index, mean ± SD		23.4 ± 2.7
Gainful employment, %		96
Educated at university level *, %		74
Income *:		
	≤25,000 SEK ** a month, %	44
	25,001–30,000 SEK ** a month, %	23
	≥30,001 SEK ** a month, %	33
Participant and both parents born in Sweden, %		83
Having a driver’s licence, %		93
Usually access to a car, %		78
Leaving home 7–9 a.m. to cycle to work, %		68
Number of bicycle-commuting trips per year ***, mean ± SD		277 ± 178
Overall physical health either good or very good, %		84
Overall mental health either good or very good, %		83

Notes: Values are based on self-reports; * Background factor used as a predictor variable in the multiple regression analyses; ** SEK = Swedish crowns/kronor, year 2005: €1 ≈ 9 SEK; US$1 ≈ 8 SEK; *** The number of bicycle-commuting trips per year is based on 920 participants. The low response rate is due to missing values in one or more of the 12 months leading to exclusion in the sum score.

#### Representativity of Participants

Active commuters constitute a minor group within the general population. It was therefore not realistic, in practical terms, to recruit an adequate number of participants from a random population sample. The bicycle participants were, nevertheless, recruited with the aim of achieving a reasonable representation of the adult active commuters in the inner urban and suburban areas of Greater Stockholm during the recruitment period. We were, however, concerned about the representativity of the advertisement-recruited participants. Therefore, we compared ratings of route environments done by advertisement-recruited bicycle commuters with ratings done by street-recruited bicycle commuters [5]. The street-recruited bicycle commuters were considered to represent the population of active commuters with better certainty than that of the advertisement-recruited bicycle commuters. In general, the results indicated a good correspondence between the ratings of the advertisement- and the street-recruited bicycle commuters. For example, ratings of the ACRES items for suburban areas of Greater Stockholm by advertisement- and street-recruited participants were assembled along the line of identity (r = 0.96).

### 2.2. The Physically Active Commuting in Greater Stockholm Questionnaire (PACS Q)

The PACS Q1 and PACS Q2 are self-report questionnaires in Swedish. They include questions about background factors of the participants and different aspects of active commuting. They comprise 35 and 68 items, respectively. The ACRES is included in the PACS Q2.

#### 2.2.1. Measures of Background Factors of Participants

Data on sex, age, weight, height, employment and number of bicycle-commuting trips per month were obtained from the PACS Q1. The body mass index (BMI) was calculated by dividing body weight by height squared (kg·m^−2^). Active commuting trips per year were calculated by adding each of the 12 months’ average trip frequency per week, dividing by 12 and multiplying by 52. Educational levels, income, ethnicity, having a driver’s license, having access to a car, time leaving home to cycle to work and overall physical and mental health were obtained from the PACS Q2 (see Table 1).

#### 2.2.2. The Active Commuting Route Environment Scale (ACRES)

The ACRES consists of 18 items for the assessment of bicyclists’ perceptions and appraisals of their self-chosen commuting route environment, which are potentially associated with active commuting [14]. In this study 16 items were used (see Table 2). The two items that were excluded in this study are termed *on the whole* and *short or long*. The reason for not using the item *on the whole*, which considers perceptions of the route environment on the whole, is that it is too general for the aim of this study. The reason for not using the item *short or long*, which considers perceptions of the entire trip distance, is that a considerable part of our participants also cycle in the inner urban areas.

Each ACRES item considers the inner urban area of Greater Stockholm, Sweden, and the suburban areas surrounding it within Stockholm County, separately. The questionnaire instructions include a drawn map that separates the two areas (see [14]). The participants were asked to differentiate between their experiences of their active commuting route environment in the inner urban area and in the surrounding suburban areas. All items have two identical parallel response lines. One line refers to the inner urban area and the other to the suburban areas (see [15]). The separation between the inner urban and suburban areas was primarily based on their constituting different environments. In this study, we only use data regarding the suburban environments.

Fifteen-point response scales ranging from 1 to 15 are used. The scales have adjectival opposites labelled, for example, *very low* and *very high*. One item constitutes an exception: the item *bicycle paths/lanes/roads* has an 11-point response scale ranging from 0% (0) to 100% (10) (see Table 2). The 15-point response scales have numbered continuous lines, *i.e.*, whole numbers from 1 to 15. In addition, number 8, as a neutral option in the middle, is labelled, for example, *neither low nor high* (see [15]).

The participants are instructed in the questionnaire to recall and rate their overall experience of their self-chosen route environments based on their active commuting to their place of work during the previous two weeks. The participants were not informed about the objective of the ACRES.

**Table 2:** The Active Commuting Route Environment Scale (ACRES) assessing bicyclists’ perceptions and appraisals.

Question	15-Point Response Scale		Variable Name
	1	15	
Do you think that, on the whole, the environment you cycle in stimulates/hinders your commuting?	Hinders a lot	Stimulates a lot	Hinders or stimulates *
How do you find the exhaust fume levels along your route?	Very low	Very high	Exhaust fumes
How do you find the noise levels along your route?	Very low	Very high	Noise
How do you find the flow of motor vehicles (number of cars) along your route?	Very low	Very high	Flow of motor vehicles
How do you find the speeds of motor vehicles (taxis, lorries, ordinary cars, buses) along your route?	Very low	Very high	Speeds of motor vehicles
How do you find other cyclists’ speeds along your route?	Very low	Very high	Speeds of bicyclists
How do you, as a cyclist, find the congestion levels in mixed traffic, caused by all types of vehicles, along your route?	Very low	Very high	Congestion: all types of vehicles
How do you find the congestion levels caused by the number of cyclists on the cycle paths/cycle lanes along your route?	Very low	Very high	Congestion: bicyclists
How do you find the occurrence of conflicts between you, as a cyclist, and other road users (including pedestrians) along your route?	Very low	Very high	Conflicts
About how large a part of your route consists of cycle paths/cycle lanes/cycle roads separated from motor-car traffic?	0%	100% **	Bicycle paths/lanes/roads
How unsafe/safe do you feel in traffic as a cyclist along your route?	Very unsafe	Very safe	Traffic: unsafe or safe
How do you find the availability of greenery (natural areas, parks, planted items, trees) along your route?	Very low	Very high	Greenery
How ugly/beautiful do you find the surroundings along your route?	Very ugly	Very beautiful	Ugly or beautiful
To what extent do you feel that your cycle trip is made more difficult by the course of the route? For example, a course with many sharp turns, detours, changes in direction, side changeovers, *etc.*	Very little	Very much	Course of the route
To what extent do you feel that your cycle trip is made more difficult by hilliness? Base this on the route to and from your place of work/study.	Very little	Very much	Hilliness
To what extent do you feel that your progress in traffic is worsened by the number of red lights during your trip to your place of work/study?	Very little	Very much	Red lights

Notes: This is a translation of the original ACRES in Swedish; * Outcome variable;** 11-point scale.

A more detailed description of the development of the ACRES and its items, as well as of its validity and reliability, has been reported elsewhere [5,14]. In brief, the ACRES was characterized by considerable criterion-related validity and reasonable test-retest reproducibility. The validity assessments were based on measured or expected differences between the inner urban and suburban areas of Greater Stockholm. The results showed, for example, a high correlation (r = 0.94) between commuters’ mean values for differences in ratings of inner urban and suburban environments and experts’ corresponding values. The test-retest assessments regarding reliability showed, for example, that intra-class correlation coefficients (ICCs) ranged from moderate (0.46) to almost perfect (0.82) for measurements of the suburban environment [14].

### 2.3. Study Area

The suburban commuting route environments studied surround the inner urban areas of Greater Stockholm, Sweden, and are located in its suburban and rural areas. The inner urban area includes the city sections of “Gamla stan” (the Old Town), Södermalm, Kungsholmen, Vasastan, Norrmalm and parts of Östermalm (Figure 1). The suburban and rural areas contain a mixture of residential areas, smaller industrial areas and managed forests, as well as agricultural land. The residential areas either comprise predominantly single houses or constitute more dense areas with multi-storey houses. The single houses were mainly built during the 1930s and onwards, in different architectural styles, whereas the majority of the densely built-up areas were fashioned in a modernistic style after the Second World War and during the 1970s. The residential density of the landscape normally varies with proximity to underground or commuter train stations, which have small centres near the stations. As an indication of residential density of the suburban parts of our study area, we have chosen the southern and westerns suburbs of the Municipality of Stockholm, and in 2005 this amounted to approximately 3500 and 2900 residents per square km, respectively [16].

Houses are generally placed as separate entities in the landscape, not in blocks, and the streets are not normally laid out in a grid-like streetscape. Instead, the main roads often follow old road networks formed during the agricultural period of the landscape.

There are trees or other forms of greenery in gardens and between the multi-storey houses, but normally not in alleys bordering the streets. The settlements, as well as the roads and traffic zones, lie in former agricultural landscapes in the sediment-filled valleys in this rift valley landscape. Between the valleys, the bedrocks often rise in faults, which are mostly covered with coniferous forest. The bedrocks often protrude from the thin soil cover (moraine). Forest-dominated areas stretch from the rural areas towards and into the centre of the region, between settlements and traffic zones, like ten green wedges. Lakes, islands and the Baltic Sea are other components.

The valleys in this area are basically flat, but the road system also includes gentle slopes of infrequent moraine hills from the deglaciation, with normally not more than 10–15 metres of elevation. Some arterial highways pass through the landscape and do so with varying contact with cyclists and pedestrians.

### 2.4. Statistical Analyses

Questionnaire data were entered in the Statistical Package for the Social Sciences and analysed in version 21.0 (IBM SPSS Inc., Somers, NY, USA). All data entered from the PACS Q2 were checked for accuracy. A small number of individuals were excluded because they stated that they had not been cycling the last two weeks, which had been requested. In addition, some participants were excluded, mainly because of incorrect or incomplete ACRES data. Participants with three or less missing ACRES values for cyclists were used for the following measures: (1) percentages and mean scores ± 1 standard deviation (SD) used to report the background factors of the participants; (2) the values of the ACRES items presented as mean scores ± 1 SD; and (3) interrelations between the variables assessed using Pearson’s correlation coefficient (r). A detailed flowchart reporting numbers and reasons for excluding PACS participants and data has been published elsewhere [17].

Simultaneous multiple regression analysis was chosen to explore associations between the outcome variable, *hinders or stimulates*, and the “background” and “environmental” predictor variables (see Table 2). The background predictors used were: *sex* (dichotomous categorical variable: females = 0 and males = 1), *age* (continuous variable), *education* (categorical variable coded as dichotomous: educated at university level = 0 and not educated at university level = 1) and *income* (categorical variable coded as three categories: ≤25,000 SEK/month = 1, 25,001–30,000 SEK/month = 2 and ≥30,001 SEK/month = 3; SEK = Swedish crowns/kronor, year 2005: ≈ 9 SEK; US$1 ≈ 8 SEK) (see Table 1). Two models were run. In Model 1, *traffic: unsafe or safe* was excluded, and in Model 2 it was included as a predictor. The reason for including *traffic: unsafe or safe*, a variable that we normally regard as an outcome variable was its possible association with the outcome variable, *hinders or stimulates*. Only participants who had no missing values for any of the variables were used in the simultaneous multiple regression analyses.

The linearity of the environmental variables was assessed visually by means of scatterplots, boxplots and error bars before running the simultaneous multiple regression analyses. All environmental variables showed reasonable linearity and were therefore used in the analyses. Before the analyses, interrelations between the environmental variables were assessed with Pearson’s correlation coefficient. The correlations between environmental predictors were, in absolute values, r ≤ 0.76 (correlations between the background variable age and the environmental predictors were, in absolute values, r ≤ 0.16), indicating no problems with multicollinearity. In addition, the variance inflation factor (VIF) was used to checked multicollinearity. Both models’ VIFs (Model 1: all values ≤ 2.99, mean: 1.76, and Model 2: all values: ≤ 2.99, mean: 1.77) indicated no problem.

Possible extreme data cases were identified using Cook’s distance. No extreme data cases were found in either of the models (Model 1: all values ≤ 0.066, mean: 0.001, and Model 2: all values ≤ 0.061, mean: 0.001). According to the sample size used, the top limit for inclusion of standardized residuals in the models was set to ± 4 SD [18]. A total of 6 individuals in both models had a standardized residual of more than −4 (Model 1, all values ≤ −4.26, and Model 2, all values ≤ −4.33). They were, however, included in the simultaneous multiple regression analyses since they were few in number, had standardized residuals close to the limit for inclusion and because the Cook’s distance did not indicate any problems.

The values from the simultaneous multiple regression analyses are presented as unstandardized coefficients B and their 95% confidence interval (CI), standardized coefficients Beta and partial correlation coefficients, as well as the R square (R²) for the overall models. The standardized coefficients Beta are used in the regression equations since the included variables represent different scales (see above and Table 2).

After the initial simultaneous multiple regression analyses including all predictors, analyses were run to test the models’ sensitivity. The first included only the significant predictors from the initial analyses (see Results, Table 5 and Table 7). The remaining analyses excluded different significant predictors from the initial analyses based on correlations above 0.7 with other predictors (see Results, Table 4). In the last analysis for Model 1, the two excluded predictors fulfil the same criteria, but represented the coefficient with the lowest standardized Beta value of each pair of correlated variables.

To indicate significance, a statistical level corresponding to at least *p* < 0.05 was used.

## 3. Results

Mean scores on all environmental variables are shown in Table 3 and their interrelations are shown in Table 4. The range for correlations (r) between the outcome variable *hinders or stimulates* and the environmental predictors was between 0.01 and 0.59. The following items had a positive correlation (*p* < 0.05) with the outcome variable; *ugly or beautiful* (r = 0.59), *greenery* (r = 0.55), *traffic: unsafe or safe* (r = 0.32), and *bicycle paths/lanes/roads* (r = 0.08). The following items had a negative correlation (*p* < 0.05) with the outcome variable; *noise* (r = −0.39), *flow of motor vehicles* (r = −0.39), *exhaust fumes* (r = −0.37), *congestion: all types of vehicles* (r = −0.33), *course of the route* (r = −0.28), *red lights* (r = −0.28), *speeds of motor vehicles* (r = −0.26), *conflicts* (r = −0.22), *congestion: bicyclists* (r = −0.19) and *hilliness* (r = −0.10). *Speeds of bicyclists* had no significant correlation with the outcome variable (r = 0.01).

**Table 3:** Participants’ ratings of environmental variables (n = 1098–1107).

Variable	Mean ± SD	15-point Response Scale	
		1	15
Hinders or stimulates	11.31 ± 2.84	Hinders a lot	Stimulates a lot
Exhaust fumes	6.72 ± 3.55	Very low	Very high
Noise	6.95 ± 3.56	Very low	Very high
Flow of motor vehicles	7.52 ± 3.95	Very low	Very high
Speeds of motor vehicles	8.40 ± 3.25	Very low	Very high
Speeds of bicyclists	8.74 ± 2.60	Very low	Very high
Congestion: all types of vehicles	5.80 ± 3.41	Very low	Very high
Congestion: bicyclists	4.72 ± 3.40	Very low	Very high
Conflicts	4.98 ± 3.53	Very low	Very high
Bicycle paths/lanes/roads	7.04 ± 2.64	0%	100% *
Traffic: unsafe or safe	11.49 ± 2.96	Very unsafe	Very safe
Greenery	11.38 ± 3.09	Very low	Very high
Ugly or beautiful	10.78 ± 2.91	Very ugly	Very beautiful
Course of the route	5.20 ± 3.49	Very little	Very much
Hilliness	6.13 ± 3.97	Very little	Very much
Red lights	3.96 ± 3.47	Very little	Very much

Notes: * Minimal value = 0 and maximal value = 10. Percentage values have been transformed into an 11-point scale; For the questions associated with the variables, see Table 2.

**Table 4:** Correlations between ratings of environmental variables (n = 1091–1107).

Variable	1	2	3	4	5	6	7	8	9	10	11	12	13	14	15	16
1. Hinders or stimulates	-															
2. Exhaust fumes	−0.37 *	-														
3. Noise	−0.39 *	0.76 *	-													
4. Flow of motor vehicles	−0.39 *	0.67 *	0.72 *	-												
5. Speeds of motor vehicles	−0.26 *	0.44 *	0.48 *	0.59 *	-											
6. Speeds of bicyclists	−0.01	0.24 *	0.23 *	0.25 *	0.26 *	-										
7. Congestion: all types of vehicles	−0.33 *	0.46 *	0.48 *	0.53 *	0.44 *	0.27 *	-									
8. Congestion: bicyclists	−0.19 *	0.38 *	0.38 *	0.38 *	0.28 *	0.37 *	0.56 *	-								
9. Conflicts	−0.22 *	0.26 *	0.30 *	0.33 *	0.29 *	0.13 *	0.50 *	0.53 *	-							
10. Bicycle paths/lanes/roads	0.08 *	0.09 *	0.14 *	0.07 *	−0.01	0.15 *	−0.02	0.11 *	−0.01	-						
11. Traffic: unsafe or safe	0.32 *	−0.27 *	−0.27 *	−0.31 *	−0.33 *	−0.08 *	−0.40 *	−0.25 *	−0.40 *	0.32 *	-					
12. Greenery	0.55 *	−0.39 *	−0.37 *	−0.36 *	−0.23 *	−0.03	−0.32 *	−0.26 *	−0.21 *	0.08 *	0.27 *	-				
13. Ugly or beautiful	0.59 *	−0.38 *	−0.37 *	−0.35 *	−0.21 *	−0.02	−0.28 *	−0.19 *	−0.15 *	−0.01	0.19 *	0.73 *	-			
14. Course of the route	−0.28 *	0.21 *	0.20 *	0.19 *	0.21 *	0.04	0.32 *	0.24 *	0.34 *	−0.07 *	−0.32 *	−0.20 *	−0.17 *	-		
15. Hilliness	−0.10 *	0.10 *	0.12 *	0.13 *	0.08 *	0.15 *	0.15 *	0.16 *	0.12 *	−0.04	−0.14 *	−0.05	−0.05	0.28 *	-	
16. Red lights	−0.28 *	0.35 *	0.34 *	0.38 *	0.30 *	0.08 *	0.38 *	0.31 *	0.34 *	0.01	−0.25 *	−0.31 *	−0.28 *	0.37 *	0.16 *	-

Note: * *p* < 0.05.

The results of the analysis for Model 1 (in which the item *traffic: unsafe or safe* was excluded as a predictor) are shown in Table 5. About 45% of the variance of the outcome variable, *hinders or stimulates*, was explained by the predictors in the model (R² = 0.440).

**Table 5:** Model 1, in which the item *traffic: unsafe or safe* was excluded: Simultaneous multiple regression analysis of route environment and background variables (n = 1056).

Outcome Variable	y-Intercept	95% CI		*p*-Value	
Hinders or stimulates	6.08	4.95–7.21		0.000	
**Predictor Variable:**	**Regression Coefficient**				**Partial Correlation Coefficient**
	**Unstandardized B**	**95% CI**	**Standardized Beta**	***p* -Value**	
**Environmental Variable**					
Exhaust fumes	−0.02	−0.08–0.04	−0.02	0.582	−0.02
Noise	−0.06	−0.13–0.00	−0.08	0.045	−0.06
Flow of motor vehicles	−0.07	−0.12–−0.01	−0.10	0.014	−0.08
Speeds of motor vehicles	0.00	−0.05–0.05	0.00	0.965	0.00
Speeds of bicyclists	0.05	−0.01–0.11	0.05	0.083	0.05
Congestion: all types of vehicles	−0.05	−0.10–0.00	−0.06	0.067	−0.06
Congestion: bicyclists	0.03	−0.02–0.08	0.04	0.259	0.04
Conflicts	−0.02	−0.06–0.03	−0.02	0.480	−0.02
Bicycle paths/lanes/roads	0.07	0.02–0.13	0.07	0.005	0.09
Traffic: unsafe or safe	‒	‒	‒	‒	‒
Greenery	0.15	0.09–0.22	0.16	0.000	0.14
Ugly or beautiful	0.36	0.29–0.42	0.36	0.000	0.30
Course of the route	−0.09	−0.13–−0.05	−0.11	0.000	−0.12
Hilliness	−0.01	−0.04–0.02	−0.01	0.579	−0.02
Red lights	0.01	−0.03–0.06	0.01	0.650	0.01
**Background Variable**					
Sex	−0.17	−0.47–0.13	−0.03	0.256	−0.04
Age	0.01	0.00–0.03	0.05	0.064	0.06
Education	−0.22	−0.53–0.09	−0.03	0.159	−0.04
Income	0.00	−0.17–0.16	0.00	0.990	0.00

Note: R² = 0.440.

The results of the sensitivity analyses for Model 1 are shown in Table 6. The regression equation, for the first analysis was: y = 6.72 + 0.37 *ugly or beautiful* + 0.17 *greenery* + (‒0.13) *course of the route* + (‒0.11) *flow of motor vehicles* + (‒0.10) *noise* + 0.09 *bicycle paths/lanes/roads* (all *p*-values ≤ 0.005, R² = 0.432).

**Table 6:** Sensitivity analyses of Model 1 (n = 1087–1091).

Outcome Variable	Predictor Variable						R²
Hinders or Stimulates	Noise	Flow of Motor Vehicles	Bicycle Paths/Lanes/Roads	Greenery	Ugly or Beautiful	Course of the Route	
y-Intercept (*p*-Value)	Regression Coefficient: Standardized Beta (*p*-Value)						
6.72 (0.000)	−0.10 (0.005)	−0.11 (0.001)	0.09 (0.000)	0.17 (0.000)	0.37 (0.000)	−0.13 (0.000)	0.432
6.47 (0.000)	-	−0.17 (0.000)	0.08 (0.001)	0.17 (0.000)	0.38 (0.000)	−0.14 (0.000)	0.428
6.39 (0.000)	−0.17 (0.000)	-	0.09 (0.000)	0.18 (0.000)	0.38 (0.000)	−0.13 (0.000)	0.429
7.28 (0.000)	−0.11 (0.002)	−0.12 (0.000)	0.10 (0.000)	-	0.49 (0.000)	−0.14 (0.000)	0.423
8.36 (0.000)	−0.12 (0.001)	−0.12 (0.000)	0.06 (0.011)	0.42 (0.000)	-	−0.14 (0.000)	0.371
7.03 (0.000)	-	−0.19 (0.000)	0.09 (0.000)	-	0.50 (0.000)	−0.15 (0.000)	0.418

The results of the analysis for Model 2 (in which the item *traffic: unsafe or safe* was included as a predictor) are shown in Table 7. 

**Table 7:** Model 2, in which the item *traffic: unsafe or safe* was included: Simultaneous multiple regression analysis of route environment and background variables (n = 1056).

Outcome Variable	y-Intercept	95% CI		p-Value	
Hinders or stimulates	4.74	3.43–6.04		0.000	
Predictor Variable:	Regression Coefficient				Partial Correlation Coefficient
	Unstandardized B	95% CI	Standardized Beta	*p*-Value	
Environmental Variable					
Exhaust fumes	−0.01	−0.07–0.04	−0.02	0.628	−0.02
Noise	−0.06	−0.12–0.00	−0.08	0.054	−0.06
Flow of motor vehicles	−0.06	−0.12–−0.01	−0.09	0.020	−0.07
Speeds of motor vehicles	0.01	−0.04–0.06	0.01	0.667	0.01
Speeds of bicyclists	0.05	−0.01–0.11	0.05	0.087	0.05
Congestion: all types of vehicles	−0.04	−0.09–0.02	−0.05	0.162	−0.04
Congestion: bicyclists	0.03	−0.03–0.08	0.03	0.320	0.03
Conflicts	0.00	−0.04–0.05	0.01	0.856	0.01
Bicycle paths/lanes/roads	0.04	−0.02–0.09	0.03	0.196	0.04
Traffic: unsafe or safe	0.11	0.06–0.17	0.12	0.000	0.12
Greenery	0.14	0.08–0.21	0.15	0.000	0.13
Ugly or beautiful	0.35	0.29–0.42	0.36	0.000	0.30
Course of the route	−0.08	−0.12–−0.03	−0.10	0.000	−0.11
Hilliness	−0.01	−0.04–0.03	−0.01	0.654	−0.01
Red lights	0.01	−0.03–0.06	0.01	0.637	0.01
**Background Variable**					
Sex	−0.20	−0.50–0.10	−0.03	0.187	−0.04
Age	0.01	0.00–0.03	0.05	0.032	0.07
Education	−0.21	−0.52–0.10	−0.03	0.181	−0.04
Income	−0.02	−0.18–0.15	−0.01	0.836	−0.01

Note: R² = 0.449.

About 45% of the variance of the outcome variable, *hinders or stimulates*, was explained by the predictors in the model (R² = 0.449). The results of the sensitivity analyses for Model 2 are shown in Table 8. The regression equation, for the first analysis was: y = 4.57 + 0.38 *ugly or beautiful* + 0.16 *greenery* + 0.13 *traffic: unsafe or safe* + (‒0.13) *flow of motor vehicles* + (‒0.11) *course of the route* + 0.06 *age* (all *p*-values ≤ 0.008, R² = 0.438).

**Table 8:** Sensitivity analyses of Model 2 (n = 1091–1093).

Outcome Variable	Predictor Variable						R²
Hinders or Stimulates	Flow of Motor Vehicles	Traffic: Unsafe or Safe	Greenery	Ugly or Beautiful	Course of the Route	Age	
y-Intercept (*p*-Value)	Regression Coefficient: Standardized Beta (*p*-Value)						
4.57 (0.000)	−0.13 (0.000)	0.13 (0.000)	0.16 (0.000)	0.38 (0.000)	−0.11 (0.000)	0.06 (0.008)	0.438
4.89 (0.000)	−0.14 (0.000)	0.15 (0.000)	-	0.48 (0.000)	−0.11 (0.000)	0.07 (0.003)	0.430
6.24 (0.000)	−0.16 (0.000)	0.12 (0.000)	0.42 (0.000)	-	−0.12 (0.000)	0.06 (0.021)	0.372

## 4. Discussion

This is, to our knowledge, one of the first exploratory studies on bicyclists’ perceptions of their self-chosen suburban commuting route environment, based on a complete spatial matching of the environment and the relevant physical activity variable, namely, bicycle commuting. The main results indicate that in suburban areas, the factors aesthetics, greenery and bicycle paths seem to be, independently of one another, stimulating factors for bicycle-commuting. On the other hand, flows of motor vehicles, noise, and low “directness” of the route seem to be hindering factors. When unsafety-safety of traffic was included as a predictor, the factor bicycle paths lost its role as a significant predictor, a result that points to the importance of bicycle paths for this appraisal.

### 4.1. The Overall Models

As mentioned before, we have previously studied the inner urban area using a similar study design as in this study [15]. This discussion will therefore partly have a comparative approach. In both models studied in the suburban areas, about 45% of the variance of the outcome variable, *hinders or stimulates*, was explained by the predictors. These overall results are very similar to those of the inner urban areas, where about 40% of the variance was explained. Some of the unexplained variance could be due to missing factors of importance or to the level of reproducibility of the scale [14].

The sensitivity analyses of the models showed that removing a single variable in the pairs of predictor variables that correlated higher than r = 0.70 had rather small effects on the R²-values of the model. Furthermore, each removal led to that the standardized Beta value of the remaining variable increased. This point to the value of future studies with the aim of understanding the more precise contribution of the single variables *ugly or beautiful* and *greenery*, as well as *noise* and *flow of motor vehicles*, respectively.

Only one of the background variable predictors, namely *age*, contributed to the variance of the outcome variable in Model 2. Although the results indicate only small effects of the background variables on the outcome variable, it seems important to include and explore them also in future analyses. A discussion of the role of the environmental predictors will follow here. We will start with the predictors that had a stimulating effect followed by those with an inhibiting effect.

### 4.2. Aesthetics and Greenery

The predictor that contributed the most to the variance of the outcome variable in both of our models was *ugly or beautiful*. This was also seen in the inner urban area [15]. Aesthetics has been indicated as a factor of more substantial importance for recreational cycling than transport cycling [19]. Furthermore, appraisals of the “aesthetic nature of the environment” have been found to be associated with walking for exercise or recreation, but not for walking for transport (for a review, see [20]). In contrast, our results demonstrate that aesthetics is stimulating for transport cycling for commuting purposes.

This finding is, however, somewhat difficult to interpret in more concrete terms since aesthetics probably is a composite variable including aspects such as architecture, water, greenery and open spaces (see below). The present as well as the previous study [15] lends support to the view that greenery plays a role in aesthetics. This is because it correlated strongly in the present study with *ugly or beautiful* (r = 0.73; see Table 4). Still, *greenery* was assessed separately in the suburban area, and just as in the inner urban area [15], it contributed in and of itself positively to the variance of the outcome variable in both our models. Previous research on the relationship between natural environments and bicycling is sparse and inconclusive, indicating the complexity and difficulties in studying these issues (for an expanded discussion, see [15]). For example, a negative relation between whether people bicycle-commuted or not and the percentage of green space within a 1-km radius around their home was found. A stated possible explanation for this finding was that destinations, such as shops, tended to be further away, making distances less bikeable in greener living environments [21].

**Figure 2:**
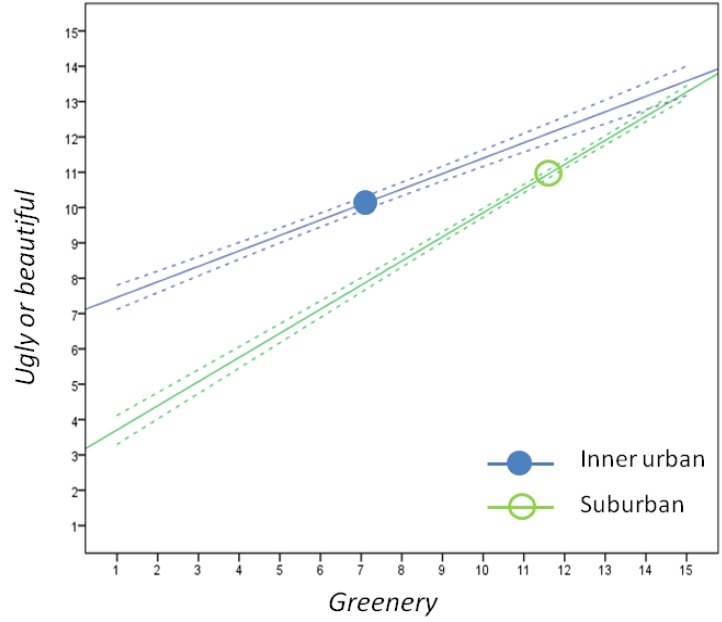
The relationship between ratings of *ugly or beautiful* and *greenery* in the inner urban and suburban areas. Y-axis: *Ugly or beautiful*: “How ugly/beautiful do you find the surroundings along your route?” (1 = very ugly and 15 = very beautiful), and x-axis: *Greenery*: “How do you find the availability of greenery (natural areas, parks, planted items, trees) along your route?” (1 = very low and 15 = very high) The upper blue lines represent the inner urban areas [15]. The lower green lines represent the suburban areas. The solid lines represent the regression lines and the dashed lines represents the 95% confidence intervals (CI) (Inner urban: y = 7.02 (6.64 – 7.41) + 0.44 (0.39 – 0.48) x, (95% CI), and suburban: y = 3.03 (2.58 – 3.47) + 0.68 (0.65 – 0.72) x). Pearson’s correlation was for: inner urban: 0.54 (n = 822); and suburban: 0.73 (n = 1104). The blue filled dot represents the mean values for *greenery* and *ugly or beautiful* in the inner urban environment (7.1 and 10.1, respectively) (*cf.* [15]). The green non-filled dot represents the corresponding values for the suburban environment (11.4 and 10.8, respectively) in the present study (see Table 3).

Figure 2 illustrates the relationship between greenery and aesthetics in the inner urban and suburban areas. If greenery was the sole ingredient of aesthetics, the regression lines would run along the line of identity, *i.e.*, the line formed by identical values on both the x and the y axes. Instead, both regression lines clearly deviate from this, as well as between each other. This signals that ingredients other than greenery also form the content of aesthetics, as well as there being a dissimilarity between constituents of aesthetics in these two areas.

In a previous analysis [5], we have shown that the ratios between the average ratings of *greenery* and *ugly* *or* *beautiful* differed substantially between the inner urban and suburban environments for different groups of both males and females. While the ratings for aesthetics were about the same, the ratings for greenery were about 60% higher in the suburban compared with the inner urban areas [5]. In Figure 2, this is illustrated with mean values from the present as well as the previous study of the inner urban areas [15]. Given these two forms of distinct differences between the ratings of *greenery* and *ugly* *or* *beautiful* for the areas, it is noteworthy that these variables still have the same role for the outcome variable *hinders or stimulates*. This indicates that these relations represent a rather robust phenomenon.

**Figure 3:**
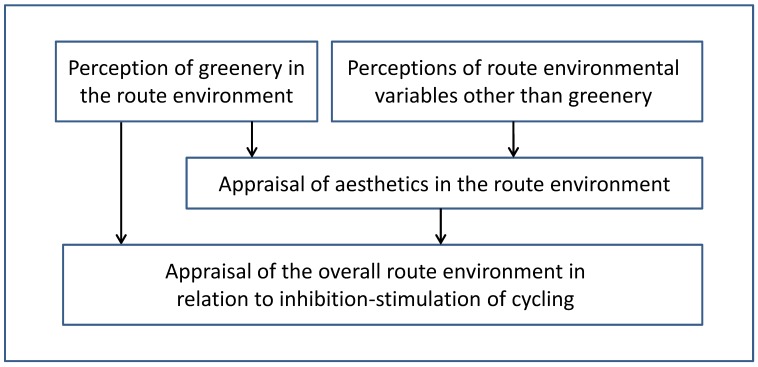
Model of relations between greenery, aesthetics and stimulation of cycling in route environments. The model illustrates that perceptions of greenery (ACRES predictor variable: *greenery*) may affect the overall appraisal of route environments (ACRES outcome variable: *hinders or stimulates*) in two ways: independently and via the appraisal of aesthetics (ACRES predictor: *ugly or beautiful*). In addition, the model illustrates that other environmental sources than greenery, such as architecture, water and open spaces, affect the appraisal of aesthetics. The model is modified from reference [23].

A more in-depth understanding of this phenomenon calls for further studies. However, before ending the discussion of this matter here, we want to summarize our understanding of it by suggesting a hypothesis that the predictor *greenery* may act in two ways on the outcome variable *hinders or stimulates*; (1) independently and (2) via the predictor variable *ugly or beautiful*. Figure 3 illustrates this hypothesis, as well as the importance of other environmental ingredients for appraisals of aesthetics. The well-established positive psychological impacts of greenery, e.g., in reducing stress (*cf.* [22]) could be a component within the independent effect of greenery on the outcome variable *hinders or stimulates.*

### 4.3. Bicycle Paths/Lanes/Roads

It seems that bicyclists in general prefer a dedicated bicycle infrastructure (for an overview, see [24]). For example, results from a stated preference study showed a preference for routes separated from motor traffic [9] and a recent route choice analysis indicated a preference for off-street bike paths [10]. The most likely reason for this preference is safety issues. The predictor *bicycle paths/lanes/roads* was also one of the predictors that contributed positively to the variance of the outcome variable in Model 1. This is reasonable. However, why this item did not contribute significantly in the inner urban area could be seen as intriguing [15]. At present we can only speculate about possible explanations. One such deals with how the item is formulated. Note that we ask about “*cycle paths/lanes/roads*”. In the inner urban area, the proportion of bicycle lanes is rather high, whereas in the suburban areas, it is very low. At the same time, bicycle paths are clearly much more preferred by bicycle commuters in Stockholm, compared to bicycle lanes [25]. Most likely, this is due to the fact that these different forms of bicycle infrastructure have different effects on the perception of traffic safety. Against this background it is more reasonable to assume that the item *bicycle paths/lanes/roads* stands out more as a stimulating factor in the suburban areas than in the inner urban areas. Along this path of thinking it is also reasonable that when *traffic: unsafe or safe* was included in our analysis as a predictor (Model 2) it took over the role of *bicycle paths/lanes/roads* as a significant predictor. *Bicycle paths/lanes/roads* can therefore be regarded as part of both the outcomes *traffic: unsafe or safe* and *hinders or stimulates*. In the inner urban setting the same phenomenon was noted for the item *congestion: all types of vehicles* [15]. Again, these interactions follow a path of reasonable roles for these items.

### 4.4. “Directness” of the Route

Connectivity and the continuity of movement of the bicycle trip are aspects that are associated with bicycle infrastructure. Greater street connectivity has been reported to be associated with physical activity (for a review of reviews, see [26]). Related to connectivity is our item *course of the route*, which indicates the level of “directness” of the route. *Course of the route* originated from the theories of space syntax (see [14]). In regards to walking, these theories stress the street network as a strong movement generator. In addition, *course of the route* could, in a broader sense, be related to street connectivity, which is one of the factors that constitute walkability (*cf.* [27]). Interestingly, connectivity, as a part of walkability, has been shown to be associated with transport bicycling [28]. *Course of the route* was one of the predictors that contributed negatively to the variance of the outcome variable in both our models. The meaning of this result is that low directness of cycle routes does not stimulate commuting cycling. The same was noted in the inner urban areas [15]. Thus, it seems that this effect of *course of the route* is independent of whether the setting is an inner urban or a suburban area.

### 4.5. Effects of Motor Vehicles

Safety aspects seem to stimulate bicycling behaviours (for an overview, see [24]) and the majority of bicyclists’ unsafety concerns are most likely to be related to motorized vehicles. For example, perceptions of “streets with a lot of car, bus and truck traffic, vehicles driving faster than 50 km/h, risk of injury from car-bike collisions, and risk from motorists who do not know how to drive safely near bicycles” were ranked among the top deterrents to cycling. On the other hand, “routes away from traffic noise and air pollution” was ranked as the strongest motivator [29]. This indicates that there are also other reasons than traffic safety for avoiding motorized traffic, reasons that have a bearing on the outcome *hinders or stimulates* cycling. In line with this, *flow of motor vehicles* and *noise* were two of the predictors that contributed negatively to the variance of the outcome variable *hinders or stimulates* (*flow of motor vehicles* in both models and *noise* in Model 1). This was not seen in the inner urban area [15]. Interestingly, *exhaust fumes* was one of the predictors that contributed negatively to the variance of *hinders or stimulates* in the inner urban area [15]. This was not seen in the suburban area. The shift in variables that indicate a negative influence on the outcome variable emphasizes the importance of studying different environmental settings separately. 

In our previous analyses, these three predictor variables, all connected to motorized traffic, were rated on average 30%–60% higher by both males and females in the inner urban areas than in the suburban areas [5]. Still, two of these variables stand out as having an inhibiting effect on commuting cycling in the suburban areas. One can only speculate on the reason for this. The findings may reflect a phenomenon whereby the effect of, for example, noise on the overall ratings of an environmental setting can be dependent on the desired expectations in relation to a certain setting (*cf.* [30]). For many people the choice of living in a suburban setting is based on a wish to avoid some environmental features of inner urban areas, such as noise. This may load the same variables with a more negative affect in suburban areas than in inner urban areas. That phenomenon has been described explicitly for noise in natural settings (e.g., [30]). Another potential cause could be that the qualitative nature of noise and the flow of motor vehicles can differ between the areas. For example, possible fluctuations in these variables in suburban areas can perhaps be viewed as being more problematic than steadier levels in inner urban areas. Furthermore, a certain flow of motorized vehicles may stand out more visually in suburban areas with more greenery and low single housing complexes than in inner urban areas dominated by massive physical entities, such as blocks with 5–7-storey-high buildings. To the best of our knowledge, these matters have not been studied before. Clearly, the influence of route environmental variables in different environmental settings deserves future studies.

### 4.6. Inner Urban vs. Suburban Areas

In summary, three items, namely, *greenery*, *ugly or beautiful* and *course of the route*, contributed to the variance of the outcome variable in both the inner urban (*cf.* [15]) and the suburban areas. Also *traffic: unsafe or safe* contributed when included (Model 2) in both areas. In the inner urban area, it took over the role of *congestion: all types of vehicles*, whereas in the suburban area it took over the role of *bicycle paths/lanes/roads*. Furthermore, *exhaust fumes* contributed in the inner urban analyses and *noise* (Model 1) and *flow of motor vehicles* in the suburban analyses (see Table 9). These findings emphasize the importance of studying different environmental settings separately. Indeed, through such analyses we can obtain an understanding of which factors seem to be important for bicycle commuting independently of the environmental setting, as well as which factors seem to be different depending on the context of the environmental setting.

**Table 9:** Environmental predictors that contributed significantly to the variance of the outcome variable *hinders or stimulates*.

Environmental Predictor	Inner Urban Area [15]		Suburban Area	
	Model 1	Model 2	Model 1	Model 2
Exhaust fumes	X	X		
Noise			X	
Flow of motor vehicles			X	X
Speeds of motor vehicles				
Speeds of bicyclists				
Congestion: all types of vehicles	X			
Congestion: bicyclists				
Conflicts				
Bicycle paths/lanes/roads			X	
Traffic: unsafe or safe	-	X	-	X
Greenery	X	X	X	X
Ugly or beautiful	X	X	X	X
Course of the route	X	X	X	X
Hilliness				
Red lights				

Notes: X = Environmental predictor that contributed significantly to the variance of the outcome variable *hinders or stimulates*; In Model 1, *traffic: unsafe or safe* was excluded as a predictor and in Model 2 it was included.

### 4.7. Strengths and Limitations

Some strengths as well as limitations regarding this study need to be mentioned and discussed. First, the advertisement recruitment strategy used could be questioned. It is difficult to use a population-based random sample when working with active commuters since they represent such a small proportion of the population. As mentioned, we were concerned about representativity and therefore, in a previous study [5], we compared environmental ratings of commuting routes between advertisement- and street-recruited bicycle commuters. The street-recruited bicyclists were thought to be more representative than the advertisement-recruited bicyclist. Overall, the two groups were in conformity. This strengthens the use of the advertisement strategy. Second, the study is based on a selected group. This could limit the generalizability. Exploring route environments using the ACRES is in a relatively early stage. Therefore, research using different environmental settings and other groups of people with different active commuting purposes and with different experiences of active commuting is of interest, not least for furthering the state of knowledge regarding behaviour changes with the aim of increasing the physical activity level. Third, this study is solely based on self-reports. There are numerous considerations to take into account when working with self-reports (*cf.* [31]). The purpose of this study was, however, to explore perceptions. Perceptions, as well as more objective aspects of the environment, seem to be important for behaviours [32]. This is obvious in relation to safety issues. For example, a road could be appraised as unsafe although it is perfectly safe in reality. Future studies that combine subjective and objective measures are of value. Fourth, the statistical approach used could be debated. We are in an initial stage working with exploring environmental factors in relation to bicycling. We did not feel that we had at the time a sufficient theoretical base to use a hierarchical approach. A future challenge is to compare the results from the regression analyses of the inner urban and suburban areas statistically.

Regardless of the possible limitations mentioned, this study has several strengths. The overall design is a strength. We study a specific group, namely bicycle commuters, and a specific environment, namely route environments. This specificity has been emphasized as being important for furthering the state of knowledge [33]. Additionally, we match the behaviour with the environment within which the behaviour occurs. The ACRES constitutes a base for this design. In addition, we study the whole commuting route, in contrast to other commonly used self-reports that consider only the neighbourhood [34,35]. Furthermore, the ACRES has, in contrast to other self-reports with fewer response alternatives (e.g., [36]), 15-point response scales. This enables other types of statistical analyses. In the case of this study, it was favourable to use a simultaneous multiple regression analysis. Also the reliability and validity assessments of the ACRES [5,14] must be regarded as a strength. Finally, studying the suburban area separately, which enables comparison with the inner urban areas, reveals important similarities as well as differences that furthers the state of knowledge.

## 5. Conclusions

In conclusion, the main results indicate that, in suburban areas, the factors aesthetics, greenery and bicycle paths seem to be, independently of one other, stimulating factors for bicycle commuting. On the other hand, flows of motor vehicles, noise, and, low “directness” of the route seem to be hindering factors. The effect of including unsafety-safety of traffic as a predictor points to the importance of bicycle paths for this appraisal. Furthermore, the results were compared with those from a similar study of an inner urban area. The comparison revealed similarities as well as differences between the areas. Thus, it is important to study different environmental settings separately. In our mind, these results constitute a basis for policy makers, urban planners and advocacy groups to consider when aiming at enhancing the route environments for bicycle commuters.
